# Circadian, Sleep and Caloric Intake Phenotyping in Type 2 Diabetes Patients With Rare Melatonin Receptor 2 Mutations and Controls: A Pilot Study

**DOI:** 10.3389/fphys.2020.564140

**Published:** 2020-10-09

**Authors:** Akram Imam, Eva C. Winnebeck, Nina Buchholz, Philippe Froguel, Amélie Bonnefond, Michele Solimena, Anna Ivanova, Michel Bouvier, Bianca Plouffe, Guillaume Charpentier, Angeliki Karamitri, Ralf Jockers, Till Roenneberg, Céline Vetter

**Affiliations:** ^1^Department of Integrative Physiology, University of Colorado Boulder, Boulder, CO, United States; ^2^Institute of Medical Psychology, Ludwig Maximilian University, Munich, Germany; ^3^Inserm UMR1283, CNRS UMR8199, European Genomic Institute for Diabetes (EGID), Institut Pasteur de Lille, University of Lille, Lille University Hospital, Lille, France; ^4^Department of Metabolism, Imperial College London, London, United Kingdom; ^5^Paul Langerhans Institute Dresden of the Helmholtz Center Munich at the Technische Universität Dresden, Dresden, Germany; ^6^Department of Biochemistry and Molecular Medicine, Institute for Research in Immunology and Cancer, Université de Montréal, Montréal, QC, Canada; ^7^School of Medicine, Dentistry and Biomedical Sciences, Wellcome-Wolfson Institute for Experimental Medicine, Queen’s University Belfast, Belfast, United Kingdom; ^8^Centre d’Études et de Recherches pour l’Intensification du Traitement du Diabète (CERITD), Sud-Francilien Hospital, Corbeil-Essonnes, France; ^9^Université de Paris, Institut Cochin, INSERM, CNRS, Paris, France

**Keywords:** MTNR1B, MT2, sleep, diet, circadian misalignment, social jetlag, diabetes

## Abstract

**Background:**

Melatonin modulates circadian rhythms in physiology and sleep initiation. Genetic variants of the *MTNR1B* locus, encoding the melatonin MT_2_ receptor, have been associated with increased type 2 diabetes (T2D) risk. Carriers of the common intronic *MTNR1B* rs10830963 T2D risk variant have modified sleep and circadian traits such as changes of the melatonin profile. However, it is currently unknown whether rare variants in the MT_2_ coding region are also associated with altered sleep and circadian phenotypes, including meal timing.

**Materials and Methods:**

In this pilot study, 28 individuals [50% male; 46–82 years old; 50% with rare MT_2_ mutations (T2D MT_2_)] wore actigraphy devices and filled out daily food logs for 4 weeks. We computed circadian, sleep, and caloric intake phenotypes, including sleep duration, timing, and regularity [assessed by the Sleep Regularity Index (SRI)]; composite phase deviations (CPD) as well a sleep timing-based proxy for circadian misalignment; and caloric intake patterns throughout the day. Using regression analyses, we estimated age- and sex-adjusted mean differences (MD) and 95% confidence intervals (95%CI) between the two patient groups. Secondary analyses also compare T2D MT_2_ to 15 healthy controls.

**Results:**

Patients with rare MT_2_ mutations had a later sleep onset (MD = 1.23, 95%CI = 0.42;2.04), and midsleep time (MD = 0.91, 95%CI = 0.12;1.70), slept more irregularly (MD in SRI = −8.98, 95%CI = −16.36;−1.60), had higher levels of behavioral circadian misalignment (MD in CPD = 1.21, 95%CI = 0.51;1.92), were more variable in regard to duration between first caloric intake and average sleep offset (MD = 1.08, 95%CI = 0.07;2.08), and had more caloric episodes in a 24 h day (MD = 1.08, 95%CI = 0.26;1.90), in comparison to T2D controls. Secondary analyses showed similar patterns between T2D MT_2_ and non-diabetic controls.

**Conclusion:**

This pilot study suggests that compared to diabetic controls, T2D MT_2_ patients display a number of adverse sleep, circadian, and caloric intake phenotypes, including more irregular behavioral timing. A prospective study is needed to determine the role of these behavioral phenotypes in T2D onset and severity, especially in view of rare MT_2_ mutations.

## Introduction

The neurohormone melatonin (5-methoxy-N-acetyltryptamine), mainly produced by the pineal gland, has multiple actions including the modulation of circadian and seasonal rhythms, sleep, and glucose regulation in mammals (Dubocovich et al., 2010; Gobbi and Comai, 2019; Karamitri and Jockers, 2019; Owino et al., 2019). Circadian melatonin production is controlled by endogenous oscillators within the hypothalamic suprachiasmatic nucleus (SCN), the biological master clock, and entrained by the environmental light-dark cycle with nocturnal peak levels (Zhao et al., 2019). Melatonin acts on two high-affinity G protein-coupled receptors, termed MT_1_ and MT_2_, which are encoded by the *MTNR1A* and *MTNR1B* genes, respectively, that are expressed in several central and peripheral tissues (Dubocovich et al., 2010; Jockers et al., 2016). Melatonin may feedback onto the SCN by activating MT_1_ and MT_2_ receptors in the SCN to regulate acute neuronal activity and clock gene expression and to phase shift and re-entrain circadian rhythms (Tosini et al., 2014). Melatonin has sleep promoting properties by acting on central melatonin receptors at different locations with distinct and most likely opposing properties of MT_1_ and MT_2_. Activation of the MT_1_ receptors are mainly implicated in the regulation of rapid eye movement (REM) sleep, whereas the MT_2_ receptors selectively increase non-REM (NREM) sleep (Gobbi and Comai, 2019).

Previous evidence from animal and human studies indicate that melatonin also modulates glucose metabolism (Van Cauter et al., 1997; Owino et al., 2019). The underlying mechanisms are not well understood, but current data indicates that melatonin can act through central and peripheral receptors to directly and indirectly modulate glucose uptake, pancreatic insulin secretion, and β-cell survival (Wehrens et al., 2017; Karamitri and Jockers, 2019). However, it is important to note that species-specific differences might exist between rodent models and humans.

Genetic studies have made important contributions to better understand the role of melatonin on glucose metabolism in humans. Genome wide association studies (GWAS) identified a frequent single nucleotide polymorphism (SNP), rs10830963G (variation of the C allele to G), in the intronic region of the *MTNR1B* gene, which was associated with increased fasting plasma glucose levels and impaired insulin secretion, as well as increased risk of T2D and gestational diabetes mellitus (Bouatia-Naji et al., 2009; Lyssenko et al., 2009; Prokopenko et al., 2009). The effect of the risk allele rs10830963 SNP probably starts early, during the development of prediabetic fasting hyperglycemia by affecting insulin secretion. The underlying molecular mechanisms are still a matter of debate (Bonnefond et al., 2016; Mulder, 2017; Garaulet et al., 2020) but might include increased expression of MT_2_ associated with stronger suppression of insulin secretion in pancreatic beta-cells (Gaulton et al., 2015). Subsequently, the association between the rs10830963 SNP with sleep timing, and the circadian system was examined by Lane et al. (2016). Based on circadian melatonin rhythm data measured in highly controlled laboratory settings, Lane and colleagues demonstrated that carriers of the *MTNR1B* risk variant rs10830963 have prolonged melatonin synthesis duration and a delayed melatonin rhythm offset in the morning hours, as compared to subjects without the risk variant. Furthermore, Lane et al. showed that T2D risk was greater among those carriers defined as “early risers” vs. “late risers.” They reasoned that because melatonin inhibits glucose-stimulated insulin secretion *ex vivo*, it is plausible that a longer duration of melatonin secretion and delayed melatonin offset in the morning hours, in concurrence with an earlier wake time, may lead to melatonin suppressing insulin secretion during times of elevated glucose intake (such as breakfast). This is in line with other research findings that suggested this might be an underlying mechanism leading to impaired glucose tolerance, which in turn results in hyperglycemia and ultimately promotes T2D (Eckel et al., 2015; Garaulet et al., 2020).

The association between the melatonergic system and T2D was further enhanced in a large-scale exon re-sequencing study of *MTNR1B* which revealed 40 non-synonymous variants of which the 36 very rare ones [minor allele frequency (MAF) < 0.1%] were significantly associated with T2D (Bonnefond et al., 2012). Functional characterization of the identified MT_2_ variants indicated that a loss of MT_2_ receptor function (LOF) was associated with T2D (Karamitri and Jockers, 2019). This conclusion was recently confirmed and further refined in terms of specific MT_2_ signaling functions (Karamitri et al., 2018). What is currently unclear is whether rare *MTNR1B* variants affect sleep and circadian behavioral phenotypes, including sleep timing, behavioral measures of misalignment, and caloric intake behavior. The aim of this pilot study was thus to complete a functional sleep and circadian phenotyping of T2D MT_2_, as these phenotypes maybe linked to elevated T2D risk. We hypothesized that patients with a rare MT_2_ mutation would exhibit unfavorable circadian, sleep, and caloric intake phenotypes as compared to T2D controls (Karamitri and Jockers, 2019).

## Materials and Methods

In this pilot case-control study, participants were followed up for a total of 4 weeks to determine whether circadian, sleep, and caloric intake patterns differ as a function T2D MT_2_ case status. Institutional review board approval was obtained from the Ludwig-Maximilian University, Munich, Germany (078-12).

### Participant Recruitment and Procedures

Recruitment was coordinated by the Centre d’Etudes et de Recherches pour I’Intensification du Traitement du Diabete (CERTID, France). Previously genotyped T2D patients with rare heterozygous *MTNR1B* variants, from the UMR8199-Lille cohort study (Bonnefond et al., 2012), living within 1.5 h of CERTID were contacted. After the exclusion of individuals with a psychiatric or neurological diagnosis, using sleep medication, or taking part in shift work, we recruited 15 subjects carrying a rare *MTNR1B* variant (Table 1 and Supplementary Table 1). Additionally, T2D control patients and non-diabetic controls, both most likely devoid of any rare *MTNR1B* variant due to their low prevalence, were recruited from the community within 1.5 h of CERTID. Ultimately, we selected 15 T2D subjects carrying a rare *MTNR1B* variant who were age and sex-matched with 15 control subjects with T2D and 15 non-diabetic controls (*N* = 45). All participants were invited to CERTID, where study materials were distributed by clinical study personnel specifically trained by C.V., N.B., and E.W. in sleep and circadian phenotyping methods. On the first day of the study, subjects completed a demographic questionnaire and obtained their wrist-worn actigraphy devices and dietary food logs. Subjects wore a wrist actigraph device (Daqtometer 2.4, Daqtix) for a total of 4 weeks, which stored 2-axial movement counts every 30 seconds. Participants indicated times when they did not wear the device in daily actigraphy logs, which were accounted for during missing data processing and data cleaning. Additionally, they were asked to fill out daily food logs. The food logs allowed participants to track all caloric intake across the 24 h period by 10 min bins. Participants indicated the start of any caloric intake and selected one or more of the six caloric variables: “meal,” “snack,” “caffeinated beverage,” “sugary beverage,” “milk,” or “alcohol.” Of the 45 subjects, two did not return food log or wrist-actigraphy data and were thus excluded from the final analytic sample (*N* = 28 diabetic participants; 50% with MT_2_ mutation; see Table 1).

**TABLE 1:** Participant characteristics and disease history by exposure group.

	**T2D (*n* = 14)**	**T2D MT_2_ (*n* = 14)**
Age (years)	66.1 (9.6)	63.4 (10.4)
Male	6 (42.9%)	8 (57.1%)
BMI (kg/m^2^)	31.2 (7.8)	33.2 (8.0)
Currently employed	2 (14.3%)	6 (42.9%)
Number of work days^a^	4.0 (1.4)	5.0 (1.3)
Work start^a,b^	08:45 (00:21)	07:58 (01:08)
Work end^a,b^	16:30 (02:48)	19:00 (02:24)
Workday alarm clock usage^a^	2 (100.0%)	5 (83.3%)
Flexible to very flexible work timing^a,c^	1 (50%)	5 (83.3)
Free-day alarm clock usage	1 (7.1%)	2 (14.3%)
Commute to work (min)^a^	25.0 (7.1)	11.0 (11.8)
Commute back home (min)^a^	25.0 (7.1)	13.5 (15.2)
Family history of diabetes	10 (71.4%)	10 (71.4%)
Year of diagnosis	1990 [1983–2001]	1995 [1983–2001]
Years since diagnosis	19.8 (6.12)	22.5 (6.21)
Impaired glucose tolerance^d^	3 (21.4%)	2 (14.3%)
Impaired fasting glucose levels^d^	3 (21.4%)	2 (14.3%)
HbA1c (%)	7.32 (1.33)	7.13 (0.68)
Year of HbA1C measurement	2014 [2013–2016]	2013 [2013–2014]
Diabetic retinopathy	3 (21.4%)	2 (14.3%)
Dyslipidemia	5 (35.7%)	6 (42.9%)
Nephropathy	0 (0.0%)	1 (7.1%)
Neuropathy	2 (14.3%)	4 (28.6%)
Microangiopathy	2 (14.3%)	1 (7.1%)
Psychiatric	0 (0.0%)	1 (7.1%)

*Data are displayed as means (±SD), median (min-max), or frequencies (%). SD, standard deviation; T2D, Type 2 diabetic control patients; T2D MT_2_, T2D patients with rare MT_2_ receptor. ^a^Amongst those who work. ^b^Data are represented as military time. ^c^Flexibility was measured on a scale of 0 being very inflexible and 3 being very flexible; here we report the number of individual who indicated that their work timing is flexible or very flexible (2 or 3). ^d^Clinically determined at study inclusion.*

### Phenotype Derivation

**FIGURE 1:**
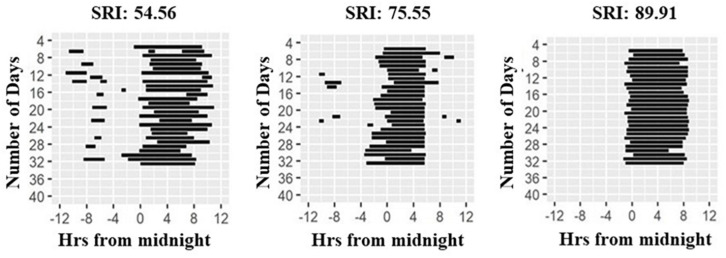
Raster plots of sleep patterns of three participants. Daily sleep duration is shown as horizontal black lines across the study period. Illustration shows individuals with highly irregular **(left panel)** to highly regular **(right panel)** sleep as quantified by the Sleep Regularity Index (SRI, Phillips et al., 2017).

Wrist-actigraphy data were used to estimate daily wake and sleep episodes using the method described in Roenneberg et al. (2015) with the ChronoSapiens software. The resulting time series was a binary distinction of sleep (0) and wake (1) in 10 min epochs. Sleep bouts that were < 2 h apart were treated as belonging to the same sleep episode. From these data, we calculated sleep onset, offset, and the midpoint of sleep (midsleep, sleep onset+ sleep offset/2), with sleep duration of the main sleep episode being the time between sleep on- and offset. Twenty-four-hour sleep duration was calculated by adding all durations of individual sleep bouts within the 24 h day. Furthermore, we computed the Sleep Regularity Index (SRI; Figure 1, Phillips et al., 2017) as a measure of sleep regularity, and Composite Phase Deviations (CPDs, Fischer et al., 2016) as a proxy for behavioral misalignment. SRI computes the likelihood that any two 10 min epochs that are 24 h apart are the same sleep/wake state, moving across the entire observation period, and ranges from 0 (highly irregular) to 100 (perfectly regular) (Phillips et al., 2017). CPD was designed to assess behavioral misalignment. It represents a high-resolution measure similar to social jetlag, both being sleep-timing based proxies for circadian misalignment. CPD can be derived based on any time-series read-out (Fischer et al., 2016) and is quantified by two vectors in this study: (i) An individual’s day-to-day difference in midsleep from 1 day to the next (y_i_), and (ii) an individual’s difference in daily midsleep times from a given reference, which we defined here as average midsleep (x_i_). Average CPD is then computed by vector length: CPD = sqrt(x_i_^2^+y_i_^2^) (Fischer et al., 2016). An advantage of this metric is that, unlike social jetlag, it can be used in both working and non-working populations, as it does not require the contrast between work and work-free days.

Using food log data, we derived the timing of participant’s first and last caloric intake, the midpoint of this caloric intake window (first caloric intake + last caloric intake/2), fasting duration (duration between last caloric intake and first caloric intake the next day), daily caloric intake frequency (the number of caloric intakes per 24 h), and snack intake frequency (the number of snack intakes per 24 h). Furthermore, we computed daily durations between the last caloric intake to sleep onset, as well as the daily duration from sleep offset to the first caloric intake. In addition, we also derived CPD for the variability of caloric intake timing (and more specifically the last caloric intake prior sleep) around an individual’s average sleep onset, which was used as the reference in the formula. This was done as evidence suggests that eating when melatonin levels are rising or high, as typically observed around sleep onset, might impair insulin sensitivity (Scheer et al., 2009; Eckel et al., 2015). Finally, we applied the same approach to determine the variability in the timing of the first caloric intake in relation to average sleep offset as the reference.

### Statistical Analyses

Potential group differences in T2D disease characteristics and severity were tested using two-sided *t*-tests for continuous variables, and chi-square tests for categorical variables. We did not have specific hypothesis regarding demographic characteristics of the two T2D patient groups and, thus, did not conduct statistical testing for these variables. We then used linear regression models to estimate mean differences and 95% confidence intervals (95% CIs) in circadian, sleep, and caloric intake phenotypes between T2D and T2D MT_2_. Model fit was assessed graphically examining residual *vs*. fitted plots. This study was initially planned as a case-control study, minimizing the influence of age and sex. However, matching was not achieved due to incomplete participant responses, so that we adjusted statistical models for age and sex to improve model fit. We did not adjust for work status; while work hours are likely to influence behavioral phenotypes, they do not affect an individual’s genotype, so that it cannot be considered a true confounder of associations between exposure group and behavioral phenotypes. In secondary analyses, we also examined mean differences in all phenotypic measures between T2D MT_2_ patients and non-diabetic controls. In sensitivity analyses, we excluded any participant with a given variable identified as an outlier (± 2 SD from the variable’s mean across all participants). A two-tailed alpha level of 0.05 as a cut-off for statistical significance for all analyses. As this is a pilot study based on a small sample size, we do not further adjust our significance threshold for multiple testing. All statistical analyses were conducted with R studio software (version 1.1463) with R version 3.4.4 (R Studio Team, 2020).

## Results

Table 1 shows the characteristics of the analytical sample. The mean age in the sample was 64.8 (SD: 9.9) years, and 50% of the 28 participants were male. There were no differences in disease severity between the two groups, as assessed by HbA1c levels (*p* = 0.71). Time since their clinical diagnosis was also similar across groups, with an average of 19.8 years for diabetic controls and of 22.5 years in T2D MT_2_ (*p* = 0.36). Three T2D and two T2D MT_2_ patients had been clinically determined as having impaired glucose tolerance and fasting glucose levels (*p* = 0.58).

**FIGURE 2:**
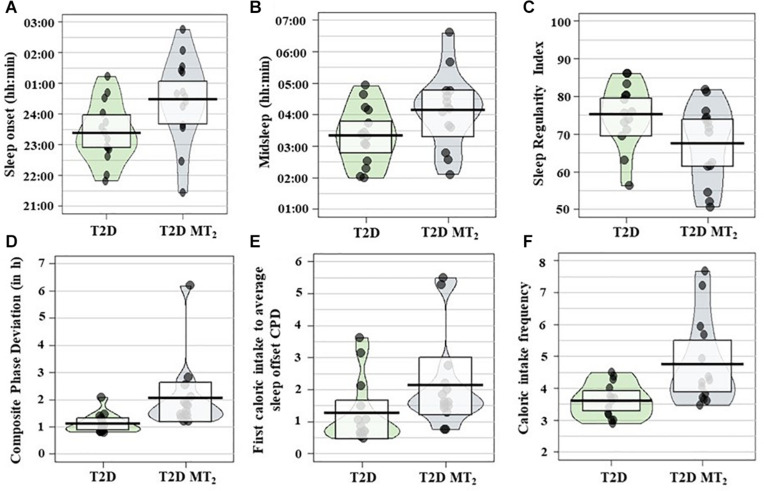
**(A)** Sleep onset, **(B)** midsleep, **(C)** Sleep Regularity Index (SRI), **(D)** Composite phase deviation (CPD), **(E)** first caloric intake to average sleep offset CPD; **(F)** caloric intake frequency; (*N* = 28) across groups. Pirate plots show median (bar), 25–75th percentile (box) and density of raw data not adjusted for age and sex. T2D controls, Type 2 diabetic control patients; T2D MT_2_, T2D patients with rare MT_2_ receptor variants.

In age- and sex-adjusted regression analyses, T2D MT_2_ had a significantly later sleep onset, 1 h and 11 min later than T2D controls (*p* = 0.01; 00:29 vs. 23:23; Tables 2, 3 and Figure2). Sleep offset was comparable across the two patient groups (7:47 vs. 7:17; *p* = 0.42). In line with these observations, T2D MT_2_ patients had a later midsleep time than T2D controls (*p* = 0.03; 4:08 vs. 3:20). T2D MT_2_ slept more irregularly with an average SRI score of 67.5, and thus 8.98 lower, and thus more irregular, than the diabetic control group (MD = −8.98, *p* = 0.03). T2D MT_2_ displayed higher levels of behavioral circadian misalignment in sleep timing, assessed by CPD (+1.21 h, *p* = 0.01). In addition, T2D MT_2_ had greater variability in the duration between first caloric intake to average sleep offset (+1.08, *p* = 0.046). While not statistically significant, T2D MT_2_ patients exhibited more variability in the duration between last caloric intake and average sleep offset (+0.89; *p* = 0.05; Table 3). T2D MT_2_ reported 1.08 caloric episodes more per 24 h period than diabetic controls (*p* = 0.02), and this difference was driven by more meal reports as snack frequency did not differ between groups. Caloric intake timing on the other hand did not show statistically or clinically meaningful differences between the two groups. Together, these findings suggest T2D MT_2_ had more caloric episodes in a 24 h day, and display greater behavioral variability in comparison to T2D controls, in addition to later timing in sleep and circadian phenotypes. Outlier correction reduced the number of participants to from 28 to 16; still, overall patterns were similar, although only group differences between sleep offset, SRI, and CPD remained statistically significant (Supplementary Tables 4,5).

**TABLE 2:** Circadian and sleep phenotypes.

	**T2D (*n* = 14)**	**T2D MT_2_ (*n* = 14)**
Twenty-four-hour sleep duration (h)	8.06 (0.96)	7.40 (1.53)
Main episode sleep duration (h)	7.90 (1.12)	7.31 (1.53)
Sleep onset^a^	23:23 (00:55)	00:29 (01:24)
Sleep offset^a^	07:17 (01:10)	07:47 (01:37)
Midsleep^a^	03:20 (00:56)	04:08 (01:11)
Sleep Regularity Index	75.3 (8.46)	67.5 (10.4)
Composite phase deviation	1.11 (0.35)	2.06 (1.29)
Fasting duration (h)	12.3 (1.01)	12.2 (1.31)
First caloric intake^a^	08:01 (00:59)	08:16 (00:58)
Last caloric intake^a^	19:42 (00:27)	20:06 (00:49)
Midpoint caloric intake window^a^	13:54 (00:35)	14:12 (00:36)
Caloric intake timing CPD	0.65 (0.58)	1.03 (0.70)
First caloric intake to average sleep offset CPD	1.27 (1.00)	2.15 (1.47)
Last caloric intake to average sleep onset CPD	3.82 (1.12)	4.60 (1.21)
Δ first caloric intake to sleep offset (h)	0.81 (0.901)	1.20 (1.46)
Δ last caloric intake to sleep onset (h)	3.65 (1.07)	4.34 (1.26)
Caloric intake frequency	3.61 (0.53)	4.75 (1.37)
Snacks intake frequency	0.55 (0.58)	0.53 (0.52)

*Data are displayed as means (± SD). SD, standard deviation; T2D, Type 2 diabetic control patients; T2D MT_2_, T2D patients with rare MT_2_ receptor variants. ^a^Military time.*

**TABLE 3:** Regression analyses of sleep and circadian phenotypes (*N* = 28).

	**T2D (*n* = 14)**	**T2D MT_2_ (*n* = 14)**	**T2D vs. T2D MT_2_. *p*-value**
Twenty-four-hour sleep duration (h)	0.00 (ref)	−0.82 (−1.84;0.20)	0.13
Main episode sleep duration (h)	0.00 (ref)	−0.76 (−1.97;0.44)	0.23
Sleep onset (h)	0.00 (ref)	1.23 (0.42;2.04)	< 0.01
Sleep offset (h)	0.00 (ref)	0.47 (−0.66;1.59)	0.42
Midsleep (h)	0.00 (ref)	0.91 (0.12;1.70)	0.03
Sleep Regularity Index	0.00 (ref)	−8.98 (−16.36;−1.60)	0.03
Composite phase deviation	0.00 (ref)	1.21 (0.51;1.92)	< 0.01
Fasting duration (h)	0.00 (ref)	0.11 (−0.83;1.05)	0.82
First caloric intake (h)	0.00 (ref)	0.41 (−0.35;1.18)	0.30
Last caloric Intake (h)	0.00 (ref)	0.38 (−0.15;0.91)	0.16
Midpoint caloric intake window (h)	0.00 (ref)	0.39 (−0.07;0.86)	0.11
Caloric intake timing CPD	0.00 (ref)	0.30 (−0.22;0.81)	0.27
First caloric intake to average sleep offset CPD	0.00 (ref)	1.08 (0.07;2.08)	0.046
Last caloric intake to average sleep onset CPD	0.00 (ref)	0.89 (0.03;1.75)	0.054
Δ first caloric intake to sleep offset (h)	0.00 (ref)	0.55 (−0.43;1.53)	0.28
Δ last caloric intake to sleep onset (h)	0.00 (ref)	0.85 (0.03;1.67)	0.052
Caloric intake frequency	0.00 (ref)	1.08 (0.26;1.90)	0.02
Snack intake frequency	0.00 (ref)	0.03 (−0.41;0.47)	0.88

*We report age- and sex-adjusted mean differences including 95% confidence intervals (95%CIs). T2D, Type 2 diabetic control patients; T2D MT_2_, T2D patients with rare MT_2_ receptor variants.*

In a secondary analyses, we also explored group differences between non-diabetic controls and T2D MT_2_. Similarly, to the comparison against T2D controls, T2D MT_2_ had a significantly later sleep onset (+1.24 h, *p* = 0.01; Supplementary Tables 2, 3) and midsleep time (+0.85 h, *p* = 0.04) in comparison to healthy controls. T2D MT_2_ displayed higher variability in their sleep timing (as assessed by CP; +.86 h, *p* = 0.046), higher levels of variability in the duration of their last caloric intake and their average time of sleep onset (+0.94 h; *p* = 0.04), and higher variability in the duration of their first caloric intake to average sleep offset (+1.00 h; 1.61 vs. 0.97; *p* = 0.046). The timing of last caloric intake to sleep onset was +0.81 h longer in T2D MT_2_ in comparison to controls (MD = +0.81, *p* = 0.04).

## Discussion

In this study, we investigated circadian, sleep, and caloric intake phenotypes of heterozygous carriers of rare MT_2_ mutations with T2D (*n* = 14) as compared to T2D controls (*n* = 14). While previous studies in humans focused mainly on the role of frequent variants in the *MTNR1B* locus, our pilot study findings provide novel impetus on the phenotypes of rare variants in the coding region of the *MTNR1B* gene. More specifically, our preliminary findings indicate that this subgroup of T2D MT_2_ patients sleep later, as evidenced by later sleep onset and midsleep times, sleep more irregularly, and show higher levels of misalignment in terms of sleep and caloric intake timing, as compared to T2D controls. From our results, we conclude that these behavioral sleep, circadian, and caloric intake phenotypes might be useful to include in future studies.

Highly controlled laboratory studies with healthy participants carrying the frequent *MTNR1B* variant rs10830963 reported an association between the rs10830963 SNP and a longer duration of elevated melatonin levels and later melatonin offset of the circadian melatonin rhythm (Lane et al., 2016). In this pilot study, at home, melatonin rhythm assessments were not feasible for logistical reasons; we therefore focused on objectively measured actigraphy data and daily food logs to quantify sleep patterns, caloric intake patterns, and proxies for circadian misalignment.

Our findings suggest that rare MT_2_ variant carriers sleep more irregularly and experience higher levels of behavioral misalignment. These carriers were younger, on average, and worked longer hours than T2D controls, which may have contributed to higher levels of behavioral circadian misalignment. In this study, misalignment was quantified based on daily sleep timing, as previously suggested by Fischer et al. (2016). The mismatch between sleep timing on work *vs*. free days (also referred to as social jetlag Wittmann et al., 2006; Roenneberg et al., 2019) has previously been associated with adverse metabolic outcomes, including obesity, insulin resistance and T2D (Wittmann et al., 2006; Roenneberg et al., 2012; Wong et al., 2015; Koopman et al., 2017), and may be a powerful tool to approximate circadian misalignment in population and field studies that do not have the possibility to assess circadian melatonin rhythms. In the laboratory setting, circadian misalignment is typically modeled by a mismatch between circadian melatonin rhythms and behavioral rhythms (Vetter, 2020). These studies have consistently shown that circadian misalignment is associated with T2D risk (Scheer et al., 2009; Eckel et al., 2015). In our study, T2D participants with a rare MT_2_ variant had higher levels of behavioral misalignment as compared to T2D and healthy controls. Our study design included T2D controls, so that despite the small sample size, it seems unlikely that the differences between groups are driven by T2D case status, and groups were comparable in terms of disease severity. This suggests that our findings identify potentially useful behavioral phenotype candidates for future studies and support the need of further in-depth phenotyping in these studies, including behavioral and physiological measures of misalignment.

**FIGURE 3:**
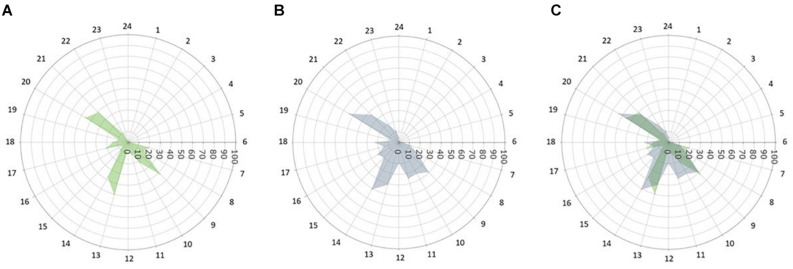
Caloric intake frequency (%) across the 24 h day. The radii of each point depict the percentage of subjects self-reporting any caloric intake by time of day (0–100, % of subjects who consumed calories at a given time). **(A)** T2D control patients, **(B)** T2D patients with rare MT_2_ receptor variants, and **(C)** Both groups overlaid.

To date, it is unclear whether and to what extent rare MT_2_ mutations affect caloric intake patterns. Our findings, based on 4-weeks of food diary entries, show that patients with rare MT_2_ variants had more frequent caloric intake episodes across the 24 h day and higher variability in duration between sleep on- and offset and the last/first caloric intake in patients with rare MT_2_ variants. This is a novel observation and adds to the existing body of evidence that tracking sleep and food timing in metabolic outcome studies might be useful. Snacks were generally uncommon amongst both groups with on average 0.55 snacks per day T2D and 0.53 snacks/day for T2D MT_2_, respectively. This is in contrast to prior reports of snacking behaviors in healthy adults, and generalizability across disease states and cultural background remain to be elucidated (Gill and Panda, 2015). Figure 3 displays caloric intake frequency in reference to the 24 h day. T2D controls have a distinct breakfast, lunch, and dinner episode, with a small percentage of subjects having a caloric episode between lunch and dinner. Similarly, a large percentage of T2D MT_2_ subjects took part in a typical breakfast, lunch, dinner, and a caloric episode between lunch and dinner. However, these peaks are not as distinct as T2D controls and are rather wide, suggesting greater variability caloric consumption. Future studies, that also have information on macro- and micronutrient composition of meals might benefit from including variability and regularity measures in their outcome quantification. Prior work suggests that dimensions beyond food composition, such as frequency and regularity, are related to cardiometabolic health (Pot et al., 2014; Hunt et al., 2020). Future work including measures of diet quality would allow further investigating the role of diet quality within the complex interplay of MT_2_ receptor variants, behavioral phenotypes, and T2D. Ideally, these studies should be prospective in nature, as this is necessary to disentangle the directionality of associations.

We also investigated a novel metric, assessing the day to day variability in last caloric intake in relation to a subject’s sleep onset. There is evidence that eating during your biological night results in metabolic dysregulation (McHill et al., 2017; Depner et al., 2018; Garaulet et al., 2020), so that a longer duration between the last caloric intake and sleep onset is considered advantageous. However, it is unclear whether consistent last caloric intake timing in relation to average sleep onset is an advantageous phenotype. In this pilot study, we examined group differences in the variability of the last caloric intake timing in relation to sleep onset. Our findings suggest that groups did not differ in terms of duration between last caloric intake and sleep onset, but patients with T2D MT_2_ had greater day-to-day variability in the timing of their first caloric intake in relation to average sleep offset. The functional relevance of this finding remains to be elucidated.

Our study has several strengths and limitations. Among the strengths, we were able to leverage an existing database of T2D patients that allowed us to recruit specifically those with rare MT_2_ mutations, and we collected objective sleep and wake data from actigraphy along with 4 weeks of diet logs. We also applied novel phenotyping methods that may generate useful references for future studies.

Our pilot study also had a number of limitations. First, the sample size was small (*n* = 28) and might not be representative of the overall population. Population-based cohort studies with larger sample sizes are needed to follow up on our results. Second, this study should be considered hypothesis-generating, rather than hypothesis-testing, and its goal was to identify potentially useful behavioral phenotypes for future research. It was not designed to identify mechanistic underpinning of disease etiology or to elucidate causal mediators of associations. Third, while we were able to comprehensively phenotype participants using continuous, objective actigraphy, future studies should include mobile or paper-based sleep logs alongside actigraphy data to further increase data quality. Fourth, daily food logs were based on self-reports only, potentially reducing the reliability of caloric intake reporting, although this bias should affect patient and control groups to a comparable extent. Future work with objective markers of diet intake, such as continuous glucose monitors, will provide further in-depth information (Knäuper et al., 2016). Fifth, it is important to note that neither healthy controls nor T2D controls were genotyped, so that it was theoretically possible for them to be carriers of a rare *MTNR1B* variant. Given the low population prevalence of the variants included in our study (MAF < 0.1% Bonnefond et al., 2014), it is unlikely that they would carry such rare mutations. In case our T2D control groups included participants with rare MT_2_ variants, though, such misclassification would result in a bias toward the null, minimizing between-group differences. Given our small sample size and the results we observed, it seems unlikely that misclassification occurred to a large extent. It is possible, though, that participants in our study are carriers of the common rs10830963 variant—which may in turn influence the circadian, sleep, and caloric intake phenotypes. In that case, however, an equal distribution of risk and non-risk variant carriers would be expected among our three study groups. It is worth mentioning that the phenotype of the common rs10830963 risk carriers is complex and not well understood at the moment, ranging from modestly increased *MTNR1B* mRNA levels to modified melatonin secretion patterns (Bouatia-Naji et al., 2009; Lyssenko et al., 2009; Prokopenko et al., 2009; Lane et al., 2016). Future studies with extensive genotyping of rare and common variants in all participants will be necessary to appropriately address genetic heterogeneity and misclassification concerns. It is also possible that the adverse phenotypes we observed are co-occurring, rather than on the causal pathway between melatonin pathways and T2D risk. Prospective study designs will help to address this limitation. Finally, our study did not include objective circadian measures, such as at home circadian melatonin rhythm assessments. Future studies should include such assessments, as they will be able to provide direct circadian phase estimates.

Taken together, our findings show that T2D patients with rare MT_2_ receptor variants display a wide range of adverse sleep, circadian, and caloric phenotypes. These phenotypes may potentially be useful to assess in future studies, together with melatonin rhythm assessments, so that the interplay between endogenous circadian melatonin rhythms, behavioral rhythms, and T2D risk can be assessed directly.

## Data Availability Statement

Data are available at: 10.25810/vs5v-yp35.

## Ethics Statement

The institutional review board approval was obtained from the Ludwig-Maximilian University, Munich, Germany (078-12). The patients/participants provided their written informed consent to participate in this study.

## Author Contributions

TR, CV, and RJ: conceptualization. CV, TR, GC, RJ, AB, PF, NB, and EW: methodology. AIm, EW, CV, NB, and TR: formal analysis. AIm and CV: writing—original draft. AIm, EW, NB, PF, AB, MS, AIv, MB, BP, GC, AK, RJ, TR, and CV: writing and review. CV, RF, and TR: supervision. All authors contributed to the article and approved the submitted version.

## Conflict of Interest

CV during the conduct of the study, received research support from the NIH and the University of Colorado Boulder, was a scientific advisory board member of Circadian Light Therapy Inc., and served as a paid consultant to the US Department of Energy outside of the submitted work. EW received research funds from the Friedrich Baur Stiftung and the Ludwig Maximilian University. BP received research funds from a Vice-Chancellor’s/Patrick G. Johnston Fellowship from Queen’s University Belfast and by a Welcome Trust Seed Award in Science (215229/Z/19/Z). The remaining authors declare that the research was conducted in the absence of any commercial or financial relationships that could be construed as a potential conflict of interest.
