# Supplementary material for: Circadian, Sleep and Caloric Intake Phenotyping in Type 2 Diabetes Patients With Rare Melatonin Receptor 2 Mutations and Controls: A Pilot Study
Source: Front Physiol. 2020 Oct 9;11:564140. doi: 10.3389/fphys.2020.564140 (PMC7583701; doi:10.3389/fphys.2020.564140)
Supplement: Supplementary file 1 [file Table_1.DOCX]

Supplementary Material

| **Supplement Table 1.** Number of subjects by specific mutation type. LOF: Loss-of-Function. | |
| --- | --- |
| **MTNR1B Gene Variant** | **Number of Subjects** |
| L60R (LOF) | 2 |
| A74T | 1 |
| I353T | 1 |
| V124I | 3 |
| R231H | 4 |
| S238G | 1 |
| A342V | 1 |
| F250V | 1 |

| **Supplement Table 2.** Circadian and sleep phenotypes. Data is displayed as means (±SD). SD: standard deviation; Controls: non-diabetic controls ; T2D MT_2_: T2D patients with rare MT_2_ receptor variants. | | |
| --- | --- | --- |
|  | **Controls (n=15)** | **T2D MT_2_ (n=14)** |
| **Twenty-four-hour sleep duration (h)** | 8.26 (0.92) | 7.40 (1.53) |
| **Main episode sleep duration (h)** | 8.23 (0.88) | 7.31 (1.53) |
| **Sleep onset^1^** | 23:18 (00:45) | 00:29 (01:24) |
| **Sleep offset^1^** | 07:32 (01:10) | 07:47 (01:37) |
| **Mid-sleep^1^** | 03:25 (00:45) | 04:08 (01:11) |
| **Sleep regularity index** | 75.9 (10.2) | 67.5 (10.4) |
| **Composite phase deviation** | 1.25 (0.53) | 2.06 (1.29) |
| **Fasting duration (h)** | 12.2 (1.00) | 12.2 (1.31) |
| **First caloric intake^1^** | 08:00 (00:55) | 08:16 (00:58) |
| **Last caloric intake^1^** | 19:48 (00:45) | 20:06 (00:49) |
| **Midpoint caloric intake window^1^** | 13:54 (00:40) | 14:12 (00:36) |
| **Caloric intake timing CPD** | 0.60 (0.30) | 1.03 (0.70) |
| **First caloric intake to average sleep offset CPD** | 1.09 (0.66) | 2.15 (1.47) |
| **Last caloric intake to average sleep onset CPD** | 3.65 (0.86) | 4.60 (1.21) |
| **Δ first caloric intake to sleep offset (h)** | 0.48 (0.62) | 1.20 (1.46) |
| **Δ last caloric intake to sleep onset (h)** | 3.48 (0.86) | 4.34 (1.26) |
| **Caloric intake frequency** | 4.19 (1.12) | 4.75 (1.37) |
| **Snacks intake frequency** | 0.49 (0.32) | 0.53 (0.52) |

1 Military Time

| **Supplement Table 3.** Regression analyses of sleep and circadian phenotypes (N=29**).** Age- and sex-adjusted mean differences including 95% confidence intervals (95%CIs). Controls: non-diabetic controls; T2D MT_2_: T2D patients with rare MT_2_ receptor variants. | | | |
| --- | --- | --- | --- |
|  | **Controls**  **(n=15)** | **T2D MT_2_ (n=14)** | **Controls *vs*. T2D MT_2_.**  ***P-value*** |
| **Twenty-four-hour sleep duration (hrs)** | 0.00 (ref) | -0.97 (-1.98;0.05) | 0.07 |
| **Main episode sleep duration (hrs)** | 0.00 (ref) | -0.96 (-2.12;0.20) | 0.12 |
| **Sleep onset (hrs)** | 0.00 (ref) | 1.24 (0.46;2.02) | <0.01 |
| **Sleep offset (hrs)** | 0.00 (ref) | 0.28 (-0.80;1.36) | 0.62 |
| **Mid-sleep (hrs)** | 0.00 (ref) | 0.85 (0.07;1.63) | 0.04 |
| **Sleep regularity index** | 0.00 (ref) | -4.39 (-11.83;3.05) | 0.26 |
| **Composite phase deviation** | 0.00 (ref) | 0.86 (0.06;1.66) | 0.046 |
| **Fasting duration (hrs)** | 0.00 (ref) | 0.02 (-0.95;0.98) | 0.97 |
| **First caloric intake (hrs)** | 0.00 (ref) | 0.32 (-0.42;1.06) | 0.41 |
| **Last caloric Intake (hrs)** | 0.00 (ref) | 0.33 (-0.26;0.93) | 0.29 |
| **Midpoint caloric intake window (hrs)** | 0.00 (ref) | 0.33 (-0.14;0.79) | 0.18 |
| **Caloric intake timing CPD** | 0.00 (ref) | 0.33 (-0.09;0.76) | 0.13 |
| **First caloric intake to average sleep offset CPD** | 0.00 (ref) | 1.00 (0.07;1.93) | 0.046 |
| **Last caloric intake to average sleep onset CPD** | 0.00 (ref) | 0.94 (0.09;1.79) | 0.04 |
| **Δ first caloric intake to sleep offset (hrs)** | 0.00 (ref) | 0.65 (-0.26;1.55) | 0.17 |
| **Δ last caloric intake to sleep onset (hrs)** | 0.00 (ref) | 0.91 (0.05;1.77) | 0.048 |
| **Caloric intake frequency** | 0.00 (ref) | 0.67 (-0.35;1.69) | 0.21 |
| **Snack intake frequency** | 0.00 (ref) | 0.17 (-0.16;0.50) | 0.33 |

| **Supplement Table 4.** Circadian and sleep phenotypes after outlier correction (±2SD). Data is displayed as means (±SD). SD: standard deviation; T2D: Type 2 diabetic control patients; T2D MT_2_: T2D patients with rare MT_2_ receptor variants. | | |
| --- | --- | --- |
|  | **T2D (n=10)** | **T2D MT_2_ (n=6)** |
| **Twenty-four-hour sleep duration (h)** | 8.13 (1.10) | 7.97 (0.35) |
| **Main episode sleep duration (h)** | 8.03 (1.20) | 8.14 (0.33) |
| **Sleep onset^1^** | 23:15 (00:59) | 00:51 (00:49) |
| **Sleep offset^1^** | 07:17 (01:14) | 07:59 (00:40) |
| **Mid-sleep^1^** | 03:16 (00:56) | 03:55 (00:44) |
| **Sleep regularity index** | 78.5 (5.39) | 69.9 (9.97) |
| **Composite phase deviation** | 1.10 (0.41) | 1.76 (0.50) |
| **Fasting duration (h)** | 12.3 (0.87) | 12.5 (0.76) |
| **First caloric intake^1^** | 07:59 (00:49) | 08:25 (00:49) |
| **Last caloric intake^1^** | 19:42 (00:30) | 19:54 (00:32) |
| **Midpoint caloric intake window^1^** | 13:51 (00:32) | 14:10 (00:35) |
| **Caloric intake timing CPD** | 0.53 (0.21) | 0.77 (0.30) |
| **First caloric intake to average sleep offset CPD** | 1.22 (0.84) | 1.47 (0.44) |
| **Last caloric intake to average sleep onset CPD** | 3.66 (1.06) | 4.26 (0.63) |
| **Δ first caloric intake to sleep offset (h)** | 0.81 (0.87) | 0.42 (0.34) |
| **Δ last caloric intake to sleep onset (h)** | 3.57 (1.09) | 3.96 (0.67) |
| **Caloric intake frequency** | 3.61 (0.53) | 4.75 (1.37) |
| **Snacks intake frequency** | 0.32 (0.31) | 0.40 (0.32) |

1 Military Time

| **Supplement table 5.** Regression analyses of sleep and circadian phenotypes after outlier correction (±2SD). We report age- and sex-adjusted mean differences including 95% confidence intervals (95%CIs). T2D: Type 2 diabetic control patients; T2D MT_2_: T2D patients with rare MT_2_ receptor variants. | | | |
| --- | --- | --- | --- |
|  | **T2D (n=10)** | **T2D MT_2_ (n=6)** | **T2D vs. T2D MT_2_.**  ***P-value*** |
| **Twenty-four-hour sleep duration (h)** | 0.00 (ref) | 0.37 (-0.54;1.28) | 0.44 |
| **Main episode sleep duration (h)** | 0.00 (ref) | 0.69 (-0.31;1.68) | 0.20 |
| **Sleep onset** | 0.00 (ref) | 0.64 (-0.43;1.70) | 0.27 |
| **Sleep offset** | 0.00 (ref) | 1.32 (0.18;2.47) | 0.04 |
| **Mid-sleep** | 0.00 (ref) | 0.98 (-0.01;1.97) | 0.08 |
| **Sleep regularity index** | 0.00 (ref) | -11.86 (-20.29;-3.44) | 0.02 |
| **Composite phase deviation** | 0.00 (ref) | 0.80 (0.27;1.33) | 0.01 |
| **Fasting duration (h)** | 0.00 (ref) | 0.81 (-0.01;1.63) | 0.08 |
| **First caloric intake** | 0.00 (ref) | 0.79 (-0.15;1.73) | 0.12 |
| **Last caloric intake** | 0.00 (ref) | 0.03 (-0.57;0.63) | 0.93 |
| **Midpoint caloric intake window** | 0.00 (ref) | 0.41 (-0.27;1.09) | 0.26 |
| **Caloric intake timing CPD** | 0.00 (ref) | 0.23 (-0.08;0.55) | 0.18 |
| **First caloric intake to average sleep offset CPD** | 0.00 (ref) | 0.11 (-0.75;0.98) | 0.80 |
| **Last caloric intake to average sleep onset CPD** | 0.00 (ref) | 0.84 (-0.19;1.88) | 0.14 |
| **Δ first caloric intake to sleep offset (h)** | 0.00 (ref) | -0.71 (-1.54;0.12) | 0.12 |
| **Δ last caloric intake to sleep onset (h)** | 0.00 (ref) | 0.61 (-0.49;1.71) | 0.30 |
| **Caloric intake frequency** | 0.00 (ref) | 0.76 (0.04;1.49) | 0.06 |
| **Snack intake frequency** | 0.00 (ref) | 0.03 (-0.34;0.40) | 0.88 |
